# Effects of Substrate and Annealing Conditions on the Ferroelectric Properties of Non-Doped HfO_2_ Deposited by RF Plasma Sputter

**DOI:** 10.3390/nano14171386

**Published:** 2024-08-25

**Authors:** Seokwon Lim, Yeonghwan Ahn, Beomho Won, Suwan Lee, Hayoung Park, Mohit Kumar, Hyungtak Seo

**Affiliations:** 1 Department of Energy Systems Research, Ajou University, Suwon 16499, Republic of Korea; what1589@naver.com (S.L.); wbumh0607@ajou.ac.kr (B.W.); dltndhks6522@ajou.ac.kr (S.L.); wq4458@ajou.ac.kr (H.P.); 2 Department of Materials Science & Engineering, Ajou University, Suwon 16499, Republic of Korea; thffmxp@gmail.com (Y.A.); mohitiopb@gmail.com (M.K.)

**Keywords:** hafnium oxide, sputtering, orthorhombic, substrate, ferroelectricity

## Abstract

In this study, the effect of annealing and substrate conditions on the ferroelectricity of undoped hafnium oxide (HfO_2_) was analyzed. Hafnium oxide was deposited on various substrates such as platinum, titanium nitride, and silicon (Pt, TiN, Si) through RF magnetron sputtering. Annealing was performed in a nitrogen atmosphere at temperatures ranging from 400 to 600 °C, and the process lasted anywhere from 1 to 30 min. As a result, it was confirmed that the orthorhombic phase, the main cause of ferroelectricity, was dominant after a post-anneal at 600 °C for 30 min. Additionally, it was observed that interface mixing between hafnium oxide and the substrate may degrade ferroelectricity. Accordingly, the highest remanent polarization, measured at 14.24 μC/cm^2^, was observed with the Pt electrode. This finding was further corroborated by piezo force microscopy and endurance tests, with the results being significant compared to previously reported values. This analysis demonstrates that optimizing substrate and annealing conditions, rather than doping, can enhance the ferroelectricity of hafnium oxide, laying the foundation for the future development of ferroelectric-based transistors.

## 1. Introduction

Currently, commercially available memory is mainly divided into volatile and non-volatile memory, represented by dynamic random-access memory (DRAM) and NAND flash memory, respectively. DRAM operates at fast write and read speeds as it stores volatile memory, while NAND flash memory is a non-volatile memory operating at slower write and read speeds and with lower power efficiency. Therefore, various next-generation memories, combining the advantages of both types, are being studied. Among them, the most representative is ferroelectric memory [1,2,3]. This type of memory uses ferroelectric material, which spontaneously separates positive and negative charges without an external electrical stimulus. Ferroelectric memory is well-known for its faster operation speed compared to conventional memory, low power consumption, and applicability to non-volatile memory due to spontaneous polarization [4,5]. There are various ferroelectric materials, with perovskite-structured oxide being the first studied [6,7]. Perovskite oxide, characterized by an ABX_3_ crystal structure, has metal ions at the center of each unit cell. When an electric field is applied, the off-center motion of these metal ions causes spontaneous polarization, making this material initially significant in ferroelectric research [6,7]. However, applying thin perovskite oxide to state-of-the-art ferroelectric devices is challenging; its ferroelectricity degrades significantly at thicknesses below tens of nanometers [8,9], rendering complex perovskite oxide inadequate for scaling down to sub-nanometer dimensions of devices [10]. Therefore, identifying an alternative ferroelectric material to perovskite oxides is crucial. As a result, several ferroelectric materials for the two-component system have been proposed, including hafnium oxide [11,12,13,14,15,16]. Widely used in semiconductors, hafnium oxide is an insulator with a high dielectric constant and stable properties in thin films. Its characteristics vary depending on the crystal structure, which generally consists of monoclinic, tetragonal, and orthorhombic phases. Usually, the monoclinic and tetragonal structures typically exhibit dielectric properties. In contrast, the orthorhombic phase, with its non-centrosymmetric structure [11], allows for spontaneous polarization of the oxygen atoms in HfO_2_ when an electric field is applied, resulting in ferroelectricity. Various deposition and post-treatment methods have been explored to induce this orthorhombic phase. Atomic layer deposition (ALD) of HfO_2_ allows for thin film formation but struggles to induce the non-centrosymmetric orthorhombic phase due to limited process control variables and a stable chemical bonding mechanism. Studies have shown that reducing the thickness of ALD-deposited HfO_2_ films enhances their ferroelectricity [12]. However, this approach also increases the risk of dielectric breakdown and leakage current in the HfO_2_ films. And pulsed laser deposition (PLD) also hard to induce the ferroelectricity of HfO_2_ with any pre- and post-treatment, like annealing [13]. Consequently, the deposition of HfO_2_ using physical vapor deposition (PVD), particularly sputtering, is being considered as an alternative method.

Compared to atomic layer deposition (ALD), sputtering offers a faster deposition rate and provides various process variables, including gas type, RF power, and working pressure. Sputtering is advantageous for controlling defect concentration, which is crucial for inducing the orthorhombic phase [15]. Due to these advantages, numerous studies have focused on fabricating ferroelectric HfO_2_ through sputtering followed by post-annealing. However, post-annealing is necessary to induce ferroelectricity in HfO_2_ deposited by sputtering, requiring a high thermal budget. For instance, a remanent polarization (2P_r_) of 12 μC/cm^2^ in HfO_2_ films is achieved after post-annealing at 1000 °C [12]. Because of these problems, co-sputtering has emerged. Co-sputtering requires a low thermal budget to induce ferroelectricity, but remanent polarization was likewise lower than that of HfO2 deposited by ALD. This is the reason for the limit on the use of ferroelectric HfO2 in advanced integrated circuit (IC) manufacturing due to its optimization with restricted thermal budgets [14].

In this study, HfO_2_ was deposited on metal substrates such as Pt and TiN, as well as on a Si substrate, using RF sputtering and then annealed by rapid thermal annealing (RTA). The electrical and ferroelectric properties of the HfO_2_ film were analyzed using a positive-up-negative-down (PUND) test, polarization hysteresis curve, piezo force microscopy (PFM), conductive atomic force microscopy (c-AFM), and endurance tests. As a result, the impact of substrate types and annealing conditions on optimizing ferroelectric properties was determined. The remanent polarization of HfO_2_ deposited on a Pt substrate and annealed at 600 °C for 30 min was measured at 14.24 μC/cm^2^, meeting the low thermal budget requirements for ferroelectricity [15]. Additionally, various analyses, including grazing incidence X-ray diffraction (GIXRD), transmission electron microscopy (TEM), and X-ray photoelectron spectroscopy (XPS), were conducted to understand the effects of fabrication process conditions on the ferroelectric characteristics of HfO_2_ thin films. These analyses confirmed that the Pt substrate had more oxygen vacancies associated with the orthorhombic phase of HfO_2_ and exhibited less interface mixing between the substrate and the HfO_2_ film. Ultimately, this study provides valuable insights into optimizing ferroelectric HfO_2_ for compatibility with conventional semiconductor devices, which could significantly impact the development of future ferroelectric devices.

## 2. Materials and Methods

First, 100 nm Pt and TiN were deposited on a 4-inch SiO_2_ substrate by e-beam evaporation and the radio frequency magnetron sputtering technique. And then, 15 nm HfO_2_ films were deposited on a Pt, TiN, low-resistive (1 × 10^−3^ Ω cm) p-Si substrate in a large area (2 in.) by radio frequency magnetron sputtering technique (Scientific Eng & Tech, Suwon, Republic of Korea). An ultrapure commercially available 2-inch hafnium oxide target (HfO_2_, 99.999%, VTM, Incheon, Republic of Korea) was used to grow the thin films. The distance between the target and the substrate holder is 40 cm. The sputtering was performed with 100 W of rf power and a working pressure of 2 mTorr. Ultrapure argon gas with a flow rate of 30 sccm and oxygen gas with a flow rate of 10 sccm were used to maintain the working pressure. Following the HfO_2_ deposition at room temperature, the rapid thermal annealing (Real RTP-100, Daegu, Republic of Korea) was performed under a nitrogen atmosphere with a flow rate of 30 sccm at 400~600 °C for 1~30 min, which led to the formation of the crystalline HfO_2_. Finally, 50-nm-thick top Pt/Au electrodes were deposited by an E-beam evaporator using a Pt/Au source (99.99%, TFN, Seoul, Republic of Korea). The crystalline nature of HfO_2_ was studied by grazing incidence X-ray diffraction (Ultima III, Rigaku, Tokyo, Japan). The piezo force microscopy (PFM) and conductive atomic force microscopy (c-AFM) measurements were performed by atomic force microscopy (MFP-3D, Oxford Instruments, Abingdon, UK) with a Pt/Ir-coated Si probe (AC240TM) probe. The PFM measurement was carried out by the PFM technique with an AC signal of 3 V/71 kHz. The c-AFM measurement was carried out by the c-AFM technique with a DC signal of 1 V. The P-V hysteresis curve and positive-up-negative-down (PUND) test measurements were performed by a probe station (SCS-4200, Keithley, Cleveland, OH, USA). The compositional analysis of HfO_2_ films was carried out using X-ray photoelectron spectroscopy (K-Alpha^+^, Thermo Fisher Scientific, Waltham, MA, USA).

## 3. Results

**Figure 1:**
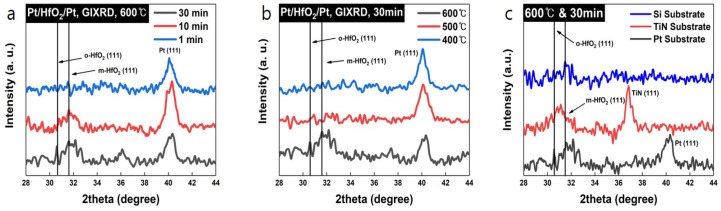
GIXRD data for (**a**) different annealing times, (**b**) different temperatures, and (**c**) different substrates with 1.5° of incidence.

Initially, the crystal structure of the HfO_2_ thin film, as influenced by the substrate type, annealing temperature, and time, was analyzed using grazing incidence X-ray diffraction (GIXRD). Figure 1a shows the GIXRD data obtained after depositing HfO_2_ on a Pt substrate and annealing it for different durations at 600 °C. In Figure 1a, peaks at 2θ = 30.33° and 31.44° were identified, corresponding to the orthorhombic and monoclinic phases of HfO_2_ [17]. The peak at 2θ = 39.82° corresponds to Pt (111) [18,19]. Additionally, an increase in the intensity of the orthorhombic phase peak was observed with longer annealing times. Figure 1b presents the GIXRD data for HfO_2_ deposited on a Pt substrate and annealed at various temperatures for 30 min, demonstrating that higher temperatures strengthen HfO_2_ crystallization. Based on these findings, HfO_2_ was deposited on Pt, TiN, and Si substrates and then annealed at 600 °C for 30 min, with the results evaluated by GIXRD. As shown in Figure 1c, HfO_2_ deposited on Si formed the dominant monoclinic phase. However, when deposited on TiN and Pt, a mixture of orthorhombic and monoclinic phases was observed. We anticipated that different lattice parameters of the substrate crystal phase could affect the HfO_2_ film phase. Also, the orthorhombic peak of HfO_2_ can overlap with tetragonal or other non-ferroelectric phases [20]. Therefore, the impact of the substrate on the crystallinity and ferroelectricity of HfO_2_, even when deposited under the same conditions, is significant, highlighting the importance of interface-controlled growth for optimizing HfO_2_ ferroelectricity.

The PUND test was initially conducted to assess the relationship between crystallinity and ferroelectricity in these thin films. As observed in Figure S3, the current in the first pulse is higher than in the second, which is attributed to the initial current required for polarization, thus confirming the ferroelectricity of the HfO_2_ film [21]. Figure S3 also shows the results of the PUND test on a Pt/HfO_2_/Pt structure device annealed at 600 °C for 30 min. It was observed that with each pulse, the difference in measured current increased.

**Figure 2:**
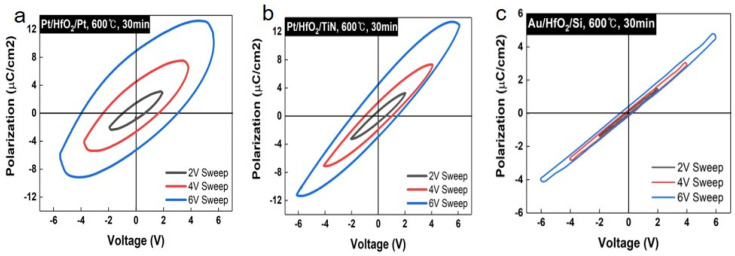
P-V hysteresis curves for HfO_2_ deposited on (**a**) Pt, (**b**) TiN, and (**c**) Si substrate.

The polarization current rose with an increasing pulse voltage. Furthermore, the remanent polarization of each device under identical annealing conditions was measured. Another voltage pulse model, shown in Figure 2a, was used to plot the polarization curve for HfO_2_ films deposited on each substrate and annealed at 600 °C for 30 min. The polarization curves of the HfO_2_ films linearly varied in the order of Pt, TiN, and Si substrates (Figure 2a,c). Similarly, the remanent polarization and coercive field at a 6 V sweep decreased in the order of Pt, TiN, and Si substrates, as shown in Table 1 and Figure S4. Additionally, for the Si substrate, the polarization loop hysteresis was notably smaller compared to other devices, which correlates with the absence of orthorhombic-phase HfO_2_, as demonstrated in the XRD data (Figure 1c), due to the lack of initial crystallization on the amorphous SiO_2_ on Si. To further explore the physicochemical relationship between the orthorhombic phase of HfO_2_ and the substrate, transmission electron microscopy (TEM) analysis and X-ray photoelectron spectroscopy (XPS) studies were conducted.

**Figure 3:**
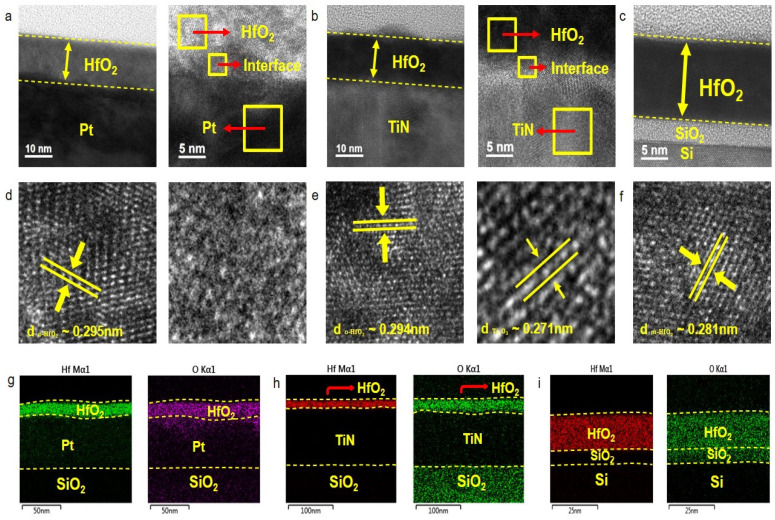
The low-resolution TEM images of the film were deposited on (**a**) Pt, (**b**) TiN and (**c**) Si and annealed at 600 °C for 30 min. The high-resolution TEM images of bulk HfO_2_ and interface between film and electrode deposited on (**d**) Pt, (**e**) TiN, and (**f**) Si. TEM-EDS elemental mapping images of HfO_2_ film deposited on (**g**) Pt, (**h**) TiN, and (**i**) Si.

The structure of HfO_2_ annealed on each substrate was analyzed using cross-sectional transmission electron microscopy (TEM). The thickness of the HfO_2_ film was consistently ~15 nm for all samples. Initially, on the Pt substrate, local chemical mixing at the interface between HfO_2_ and Pt was observed, with the amorphous structure of this interface confirmed by TEM imaging. In the bulk HfO_2_ film, the orthorhombic phase was identified, with an interplanar spacing of 0.295 nm, matching the (111) orthorhombic HfO_2_ phase [22,23]. Figure 3g showed oxygen atom diffusion beyond the HfO_2_ layer into the topmost part of the Pt electrode, leading to the partial generation of oxygen vacancies in HfO_2_, as evidenced in the supporting information (Figure S1). For the TiN substrate, the (111) orthorhombic phase of bulk HfO_2_, with the same interplanar spacing as the Pt substrate, was confirmed. However, the interface displayed a clear crystal structure between HfO_2_ and TiN. The measured interplanar distance at the interface was 0.271 nm, consistent with Ti_2_O_3_ [24]. Figure 3h revealed a wider distribution of oxygen atoms compared to Hf atoms in the HfO_2_ film, indicating interface mixing between the HfO_2_ film and TiN substrate, corroborated by the presence of Ti_2_O_3_ as identified in TEM analysis. In contrast, when HfO_2_ was deposited on the Si substrate, unlike in the other cases, interface mixing was not observed (see Figure 3c,i). The bulk HfO_2_ demonstrated a clear crystal structure with an interplanar spacing of 0.281 nm, corresponding to monoclinic HfO_2_ and aligning with the GIXRD results (see Figure 1c) [25]. TEM-EDS images confirmed that the substrate influences the bulk and interface crystal structures of HfO_2_ after annealing. Notably, while bulk HfO_2_ films on Pt and TiN substrates were primarily in the orthorhombic phase, the remanent polarization values differed by about threefold.

**Figure 4:**
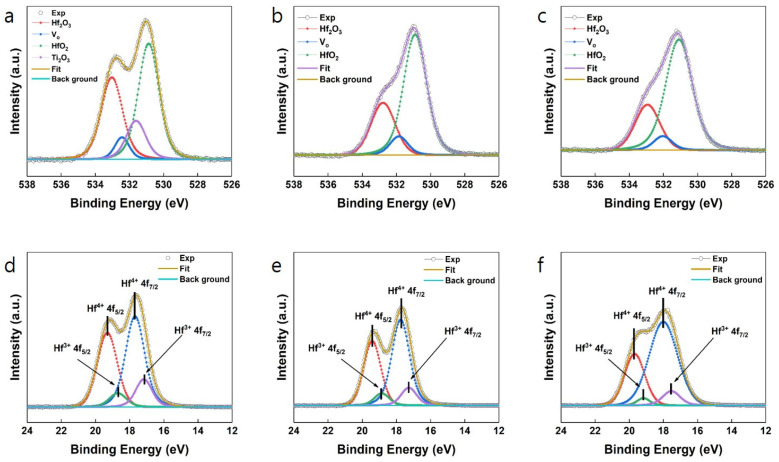
O 1s spectra for HfO_2_ deposited on (**a**) Pt, (**b**) TiN, and (**c**) Si. Hf 4f spectra for HfO_2_ deposited on (**d**) Pt, (**e**) TiN, and (**f**) Si.

X-ray photoelectron spectroscopy (XPS) analysis was conducted to investigate the bonds and defects in HfO_2_, providing further evidence for the origin of different polarization properties. Figure 4 presents the O 1s and Hf 4f binding energy spectra of HfO_2_ deposited on each substrate. The O 1s spectra reveal the presence of oxygen vacancies and sub-hafnium oxide (Hf_2_O_3_ or Hf^3+^), with peak area fractions ranging from 24 to 33%. Notably, the subpeak corresponding to Ti_2_O_3_ at 531.61 eV was identified in HfO_2_ deposited on a TiN substrate. This finding suggests the formation of Ti_2_O_3_ (even with partial crystallization) at the interface between TiN and HfO_2_, as shown in Figure 3e, resulting from oxygen supply to TiN during HfO_2_ deposition. In the Hf 4f spectra, the presence of both Hf^4+^ and Hf^3+^ binding energy peaks was observed in all samples, aligning with the subphase O species bound to Hf^4+^ and Hf^3+^ in the O 1s spectra. The increased amount of Hf^3+^, indicative of oxygen vacancy formation, is attributed to the reduction of HfO_2_ during post-deposition annealing in an N_2_ gas ambience.

Table 2 summarizes the binding energy positions and relative bonding ratios from the O 1s spectra. The main peaks for all samples are positioned between 531.08 eV and 530.87 eV, corresponding to HfO_2_ [26,27,28,29,30] (Figure 4a–c). Various dominant peaks were observed at 531.85 eV, 532.43 eV, and 532.03 eV, corresponding to oxygen vacancies on different substrates. Additionally, peaks at 532.78 eV, 533.02 eV, and 532.93 eV correspond to Hf_2_O_3_, and a peak at 531.61 eV corresponds to Ti_2_O_3_, varying with the substrate [26,27,28,29,30]. The oxygen bonding ratio was quantitatively calculated from the XPS of the O 1s spectra, revealing that in all samples, the HfO_2_ bonding ratio was the highest, followed by Hf_2_O_3_ and oxygen vacancies. Specifically, in the case of TiN, the HfO_2_ bonding ratio was lower due to Ti_2_O_3_. Additionally, the oxygen vacancy ratio was highest in the order of Pt, TiN, and Si. The previous crystal structure and remanent polarization value analyses (see Figure 1 and Figure 2) confirmed that HfO_2_′s ferroelectricity is significantly influenced by oxygen vacancies. Moreover, the interface between the substrate and HfO_2_ also impacts ferroelectricity, as seen in Figure 3c, where the native SiO_2_ layer on a Si substrate may hinder the initial spontaneous polarization of ferroelectric HfO_2_. Table 2 shows that the oxygen vacancy ratio is very similar between Si and TiN substrates, yet the remanent polarization is about 5.5 times higher with the TiN substrate. Comparing the Pt and TiN substrates, a higher oxygen vacancy ratio was observed after annealing HfO_2_ on the Pt substrate, accompanied by localized interface mixing with its amorphous crystal structure. Since Pt is less reactive, interface mixing and crystallization are less likely to occur, unlike TiN, which is more susceptible to oxidation, leading to interface mixing and the crystallization of Ti_2_O_3_. Ti_2_O_3_, having much higher resistivity than Pt, acts as an insulator similar to SiO_2_. Also, oxygen migration by annealing can induce the metastable orthorhombic phase of the HfO_2_ film. Overall, these findings indicate that interfacial properties significantly affect ferroelectricity.

Furthermore, Table 3 provides information about binding energy positions and relative bonding ratios from the Hf 4f spectra. The main doublets of Hf^4+^ 4f_5/2_–Hf^4+^ 4f_7/2_ and two sub-doublets of Hf^3+^ 4f_5/2_–Hf^3+^ 4f_7/2_ were identified. The Hf^4+^ 4f_5/2_ peaks around 19.34 to 19.57 eV, separated by 1.6 eV from the Hf^4+^ 4f_7/2_ peak at 17.74 to 17.94 eV, originating from stoichiometric HfO_2_ [31,32,33]. The Hf^3+^ 4f_5/2_ peaks around 18.76 to 18.98 eV, separated by 1.6 eV from the Hf^3+^ 4f_7/2_ peak at 17.14 to 17.34 eV, are indicative of HfO_x_ (x < 2) [33]. Table 2 and Table 3 confirm that the bonding ratios of HfO_2_ and Hf_2_O_3_ align with the calculated results from the O 1s and Hf 4f XPS spectra, allowing for analysis of the relationship between HfO_2_′s ferroelectricity and its atomic bonding structure through XPS analysis.

**Figure 5:**
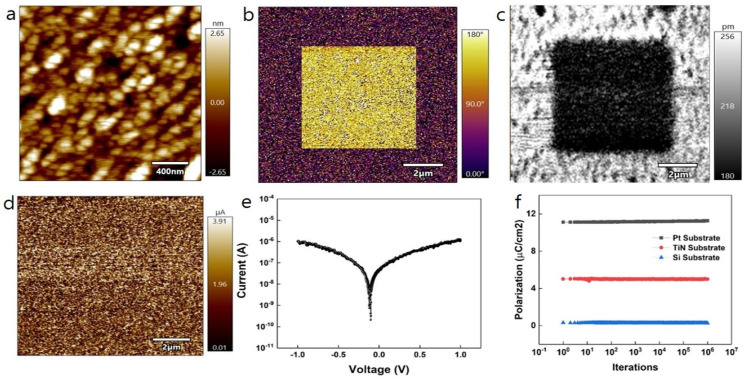
(**a**) AFM image of the film surface, (**b**) PFM phase image with tip bias, (**c**) PFM amplitude image with tip bias, and (**d**) c-AFM image with tip bias of HfO_2_ deposited on Pt substrate. (**e**) local I-V measured by c-AFM, and (**f**) endurance test for HfO_2_ film deposited on each substrate.

In Figure 5, the physical characteristics of HfO_2_, aimed at its suitability for nanoscale ferroelectric devices, were analyzed using atomic force microscopy (AFM), piezo force microscopy (PFM), and conductive atomic force microscopy (c-AFM). Additionally, an endurance test for polarization was conducted. Figure 5a presents an AFM image showing the surface roughness of HfO_2_ deposited on Pt, scanned over a 2 × 2 μm^2^ area. The root mean square (RMS) roughness, calculated from the AFM data, was 1.261 nm. Given that the average thickness of HfO_2_ is 15 nm, this indicates a relatively low surface roughness, even for films deposited by RF sputtering. The ferroelectricity of the HfO_2_ surface was then assessed with PFM. As depicted in Figure 5b,c, a 10 × 10 μm^2^ area was initially scanned without applying a bias to the probe tip. And then a 6 × 6 μm^2^ area was scanned by applying a +3 V bias to confirm the phase change. Because polarization direction is expressed by the color in the scale bar near the PFM image, phase change can be confirmed by the color of the scanned area. This resulted in a 180° phase change, indicating a complete reversal in the polarization direction of the surface, thereby confirming the potential of HfO_2_ in ferroelectric devices due to its effective polarization-switching capabilities [12,17,34]. Lastly, the I-V characteristics were measured using c-AFM. Figure 5d illustrates c-AFM scanning over a 10 × 10 μm^2^ area with a +1 V tip bias.

We confirmed the leakage current map at the level of the μA scale in the corresponding image. As a result of calculating the average scanning current based on the data of the corresponding image, 1.97 μA was obtained, and the leakage current for 1 V sweeping at a specific point, the μA level of current was also confirmed (Figure 5e). Finally, the endurance test of HfO_2_ was conducted for each substrate. First, the charge is measured for each positive and negative pulse based on the voltage pulse model of the PUND test (Figure S3a). Next, the measured charge was calculated to obtain remanent polarization per 1 cycle, and then it was measured for each cycle. As a result, it was confirmed that the remanent polarization was maintained even after repeating 1 million cycles in all samples without noticeable degradation of polarization (Figure 5f). In addition, the magnitude of the remanent polarization depending on the substrate kind coincides with that in the P-V hysteresis data. As a result, the applicability of HfO_2_ to the ferroelectric device could be confirmed in various ways through a series of experiments, as described above.

## 4. Conclusions

We have deposited the ferroelectric HfO_2_ thin film on various substrates using RF magnetron sputtering and post-anneal-induced crystallization. The ferroelectric characteristics were revealed by P-V hysteresis, PUND measurement, and local piezo force microscopy. The in-depth atomic structure and crystal analysis were performed by TEM, XPS, and GIXRD. The orthorhombic phase revealed the ferroelectricity of HfO_2_ was dominant at 600 °C and the 30-min annealing condition was confirmed by GIXRD. Based on this result, the ferroelectricity of HfO_2_ annealed at 600 °C, and 30 min was confirmed through the PUND test. The remanent polarization of HfO_2_ was higher in the case of Pt (14.24 μC/cm^2^) than in other substrates (TiN, Si) at the same annealing condition. As a result of TEM, the orthorhombic phase of HfO_2_ deposited on Pt and TiN substrate was confirmed, but the monoclinic phase of HfO_2_ deposited on Si was confirmed, and this was similar to the result of GIXRD. In addition, TEM analysis revealed that when HfO_2_ was deposited on each substrate, local chemical mixing occurred at the interface between HfO_2_ and substrate, and then the HfO_2_ phase was different depending on the substrates. The mixed phase of HfO_2_ showed the inferior initial spontaneous polarization of ferroelectric HfO_2_. From XPS measurements, the oxygen vacancy was highest in the order of Pt, TiN, and Si substrates. The orthorhombic phase of HfO_2_ leading to the relatively higher oxygen vacancy was manifested by GIXRD and TEM. The overall observation of the data suggests that the initial growth of HfO_2_ film was considerably affected by the different substrate effects to split its phase either orthorhombic-dominant or monoclinic-dominant, which is directly correlated to the degree of oxygen vacancy formation. This study therefore represents not only a process control and mechanism for ultra-thin ferroelectric HfO_2_ films requiring a low thermal budget to anneal, but also a cornerstone for applications including polarization-based memory and ferroelectric-based transistors.

## Figures and Tables

**Table 1:** In 6V voltage-sweeping remanent polarization (2P_r_) and absolute coercive field (Ec) data of HfO_2_ film deposited on Pt, TiN, and Si substrates after annealing at 600 °C for 30 min.

Samples	Remanent Polarization (2P_r_) (μC/cm^2^)	Coercive Field (V)
Pt	14.24 (±0.01)	4.03
TiN	7.43 (±0.01)	1.89
Si	0.88 (±0.01)	0.58

**Table 2:** Binding energy and relative bond fraction in the O 1s spectra of HfO_2_ deposited on different substrates.

Samples	Binding Energy (eV)			
	Hf_2_O_3_	Oxygen Vacancy	HfO_2_	Ti_2_O_3_
Pt	532.78	531.85	530.9	-
TiN	533.02	532.43	530.87	531.61
Si	532.93	532.03	531.08	-
**Samples**	**Relative binding ratio (%)**			
	**Hf_2_O_3_**	**Oxygen vacancy**	**HfO_2_**	**Ti_2_O_3_**
Pt	27.71	7.76	64.53	-
TiN	33.51	6.1	45.99	14.4
Si	24.9	6	69.1	-

**Table 3:** Binding energy and relative bond fraction in Hf 4f spectra of HfO_2_ deposited on different substrates.

Sample	Binding Energy (eV)			
	Hf^4+^ 4f_5/2_	Hf^4+^ 4f_7/2_	Hf^3+^ 4f_5/2_	Hf^3+^ 4f_7/2_
Pt	19.34	17.74	18.76	17.14
TiN	19.55	17.87	18.98	17.34
Si	19.57	17.94	18.78	17.18
**Samples**	**Relative binding ratio (%)**			
	**Hf^4+^**		**Hf^3+^**	
Pt	69.67 (±0.01)		30.33 (±0.01)	
TiN	71.79 (±0.01)		28.21 (±0.01)	
Si	84.09 (±0.01)		15.91 (±0.01)	

## Data Availability

Data are contained within the article and supporting information.

## Supplementary Materials

The following supporting information can be downloaded at: https://www.mdpi.com/article/10.3390/nano14171386/s1, Figure S1. The method of electrical and ferroelectric properties measurement. Figure S2. TEM-EDS data. (a,b,c) EDS-area & line data of Pt, TiN, Si substrate for each element and atomic percent. Figure S3. PUND test and Polarization current for Pt/HfO_2_/Pt device after annealed 600 °C and 30 min. (a) Bias pulse wave setup for PUND test. (b) Current vs. pulse time plot of PUND test for Pt/HfO_2_/Pt device after annealed 600 °C for 30 min. Figure S4. Ferroelectric Properties for each device (a) Bias pulse wave setup for drawing polarization curve. (b) Remanent polarization (2Pr) and (c) coercive field (Ec) data of HfO_2_ film deposited on Pt, TiN, Si substrate at 6 V sweeping. Figure S5. FFT images The low-resolution FFT images of local point in bulk HfO_2_ film deposited on (a) Pt, (b) TiN, (c) Si and annealed at 600 °C for 30 min. Figure S6. I–V Curve Representative switching current of HfO_2_ film deposited on Pt Substrate and annealed at 600 °C for 30 min. Table S1. XPS information for HfO_2_ film deposited on Pt, TiN, Si and annealed at 600 °C for 30 min.
