# Supplementary material for: Effects of Substrate and Annealing Conditions on the Ferroelectric Properties of Non-Doped HfO2 Deposited by RF Plasma Sputter
Source: Nanomaterials (Basel). 2024 Aug 25;14(17):1386. doi: 10.3390/nano14171386 (PMC11396947; doi:10.3390/nano14171386)
Supplement: Supplementary file 1 [file nanomaterials-14-01386-s001.zip › nanomaterials-3082908-supplementary.pdf]

Supporting Information

Effects of Substrate and Annealing Conditions on the Ferroelectric Properties of Non-Doped HfO_2 Deposited by RF Plasma Sputter

Seokwon Lim [1], Yeonghwan Ahn [2], Beomho Won [1], Suwan Lee [1], Hayoung Park [1], Mohit Kumar [2] and Hyungtak Seo [1,2,*]

[1] Department of Energy Systems Research, Ajou University, Suwon 16499, Republic of Korea; what1589@naver.com (S.L.); wbumh0607@ajou.ac.kr (B.W.); dltndhks6522@ajou.ac.kr (S.L.); wq4458@ajou.ac.kr (H.P.)

[2] Department of Materials Science & Engineering, Ajou University, Suwon 16499, Republic of Korea;
thffmxp@gmail.com (Y.A.); mohitiopb@gmail.com (M.K.)

* Correspondence: hseo@ajou.ac.kr

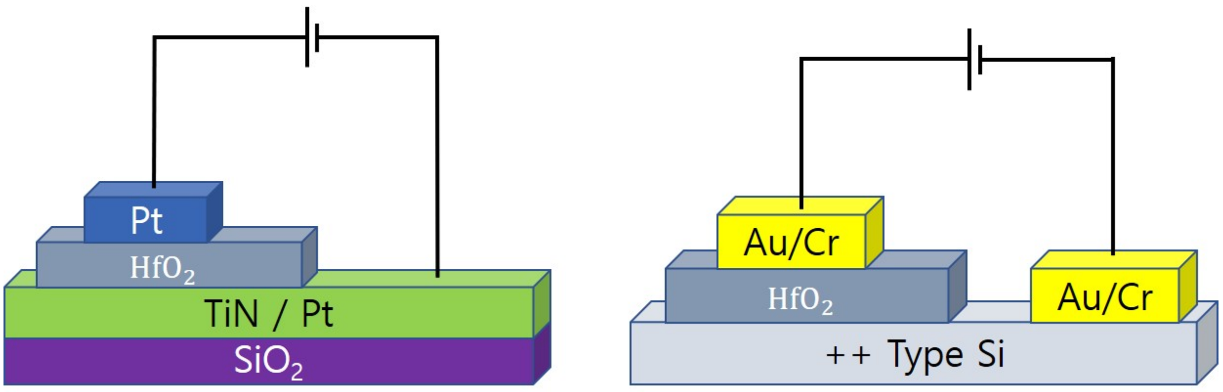

Figure S1. The method of electrical and ferroelectric properties measurement.

(a)

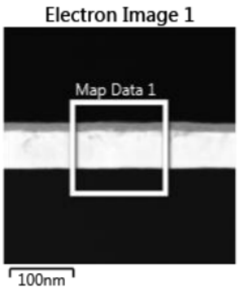

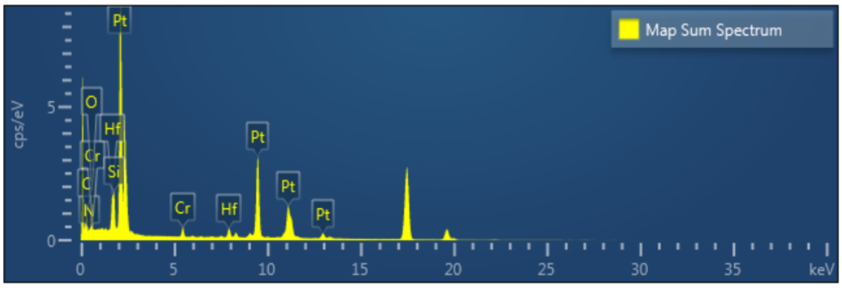

Element	Line Type	k factor	Absorption Correction	Wt%	Wt% Sigma
C	K series	3.11489	1.00	4.80	0.11
N	K series	1.80425	1.00	0.03	0.05
O	K series	1.45493	1.00	4.07	0.06
Si	K series	1.00000	1.00	3.27	0.06
Cr	K series	1.14215	1.00	1.77	0.04
Hf	L series	2.44947	1.00	7.01	0.13
Pt	L series	2.75544	1.00	79.04	0.18
Total:				100.00	

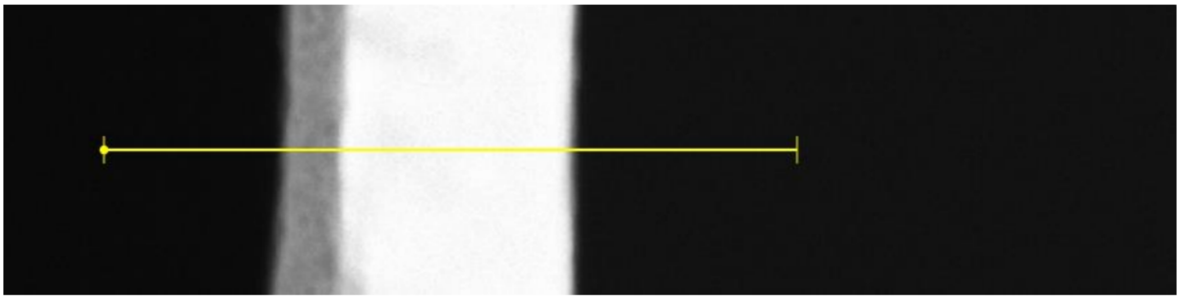

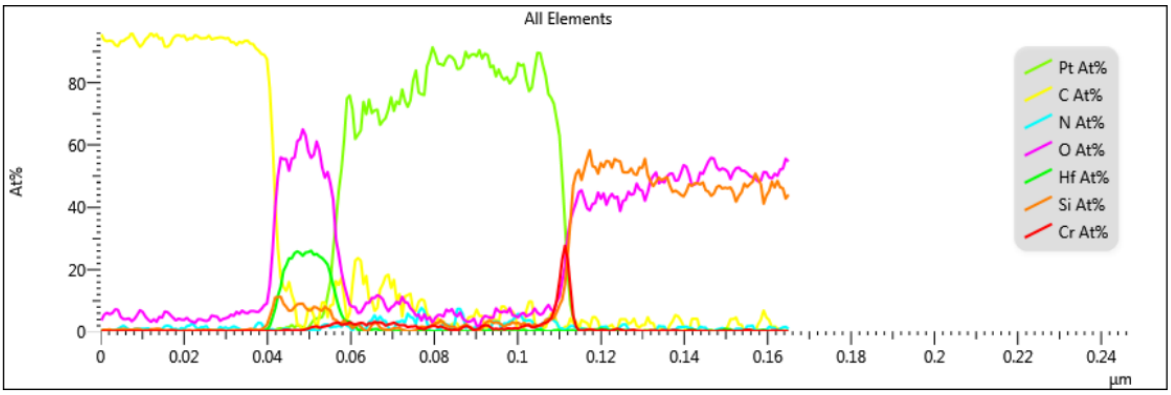

(b)

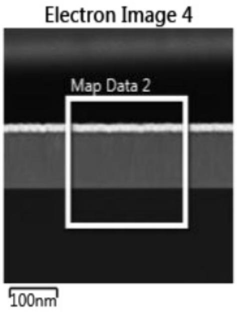

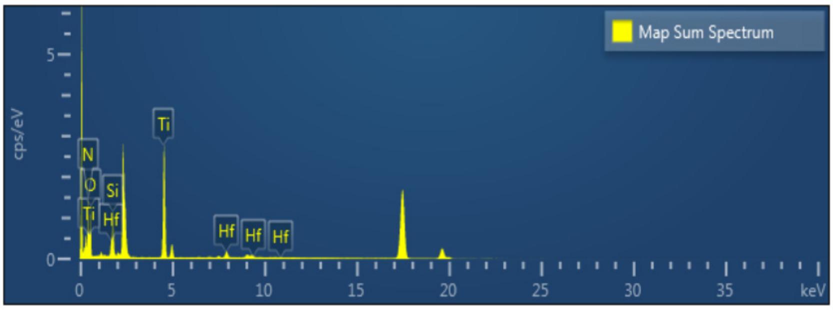

Element	Line Type	k factor	Absorption Correction	Wt%	Wt% Sigma	Atomic %
N	K series	1.80425	1.00	12.57	0.36	27.32
O	K series	1.45493	1.00	14.19	0.20	26.99
Si	K series	1.00000	1.00	11.33	0.16	12.28
Ti	K series	1.09006	1.00	49.14	0.32	31.23
Hf	L series	2.44947	1.00	12.78	0.31	2.18
Total:				100.00		100.00

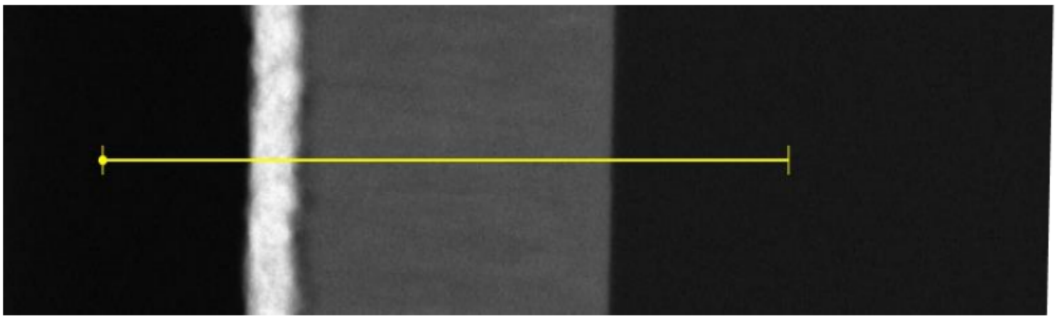

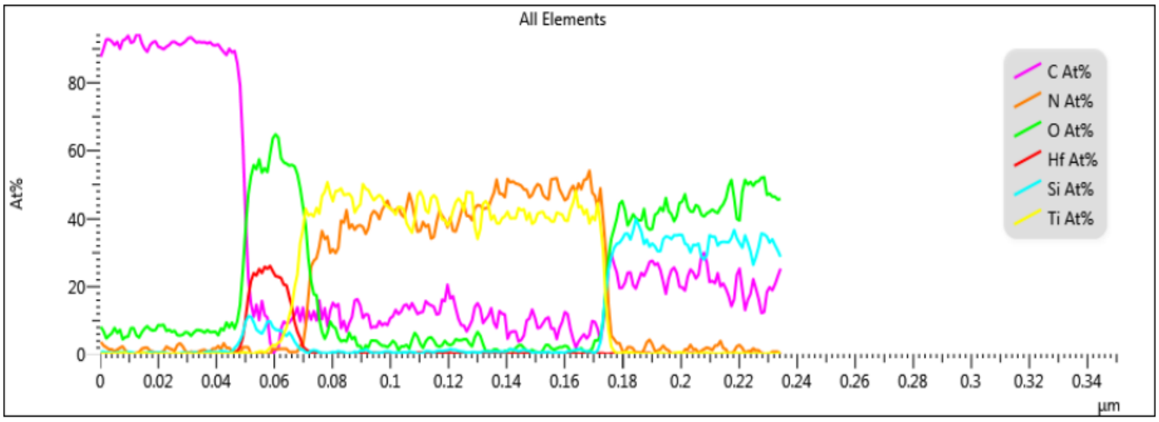

(c)

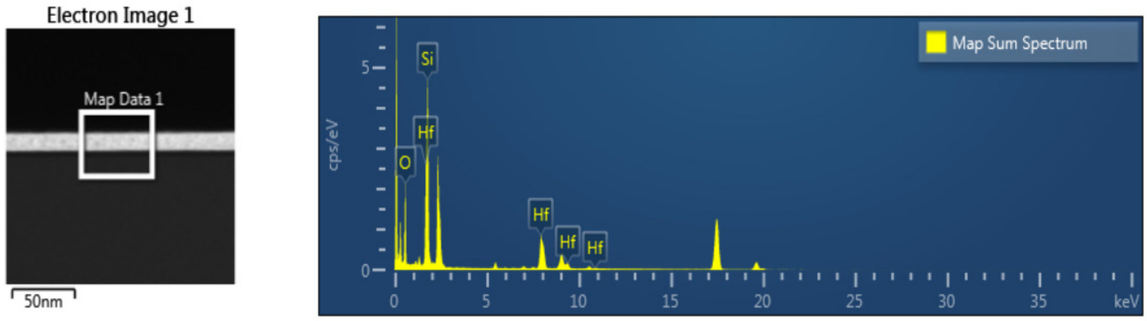

Element	Line Type	k factor	Absorption Correction	Wt%	Wt% Sigma	Atomic %
O	K series	1.45493	1.00	19.37	0.25	43.65
Si	K series	1.00000	1.00	37.04	0.35	47.54
Hf	L series	2.44947	1.00	43.59	0.44	8.80
Total:				100.00		100.00

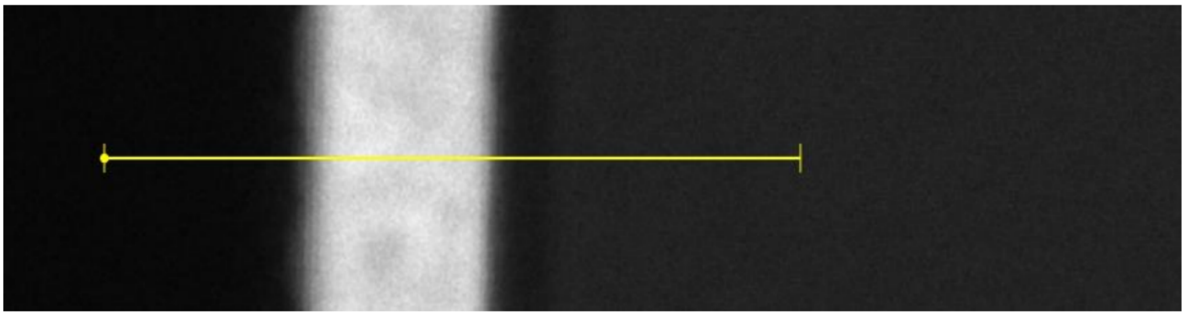

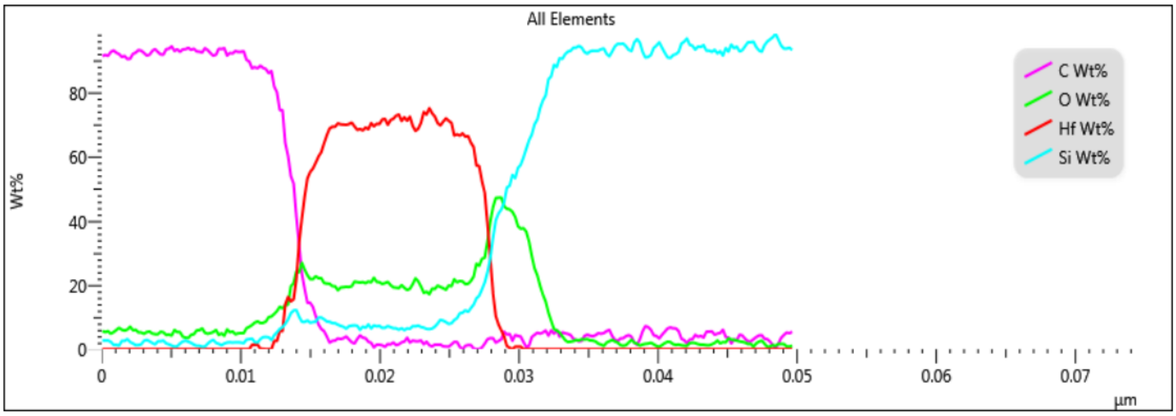

Figure S2. TEM-EDS data. (a,b,c) EDS-area & line data of Pt, TiN, Si substrate for each element and atomic percent

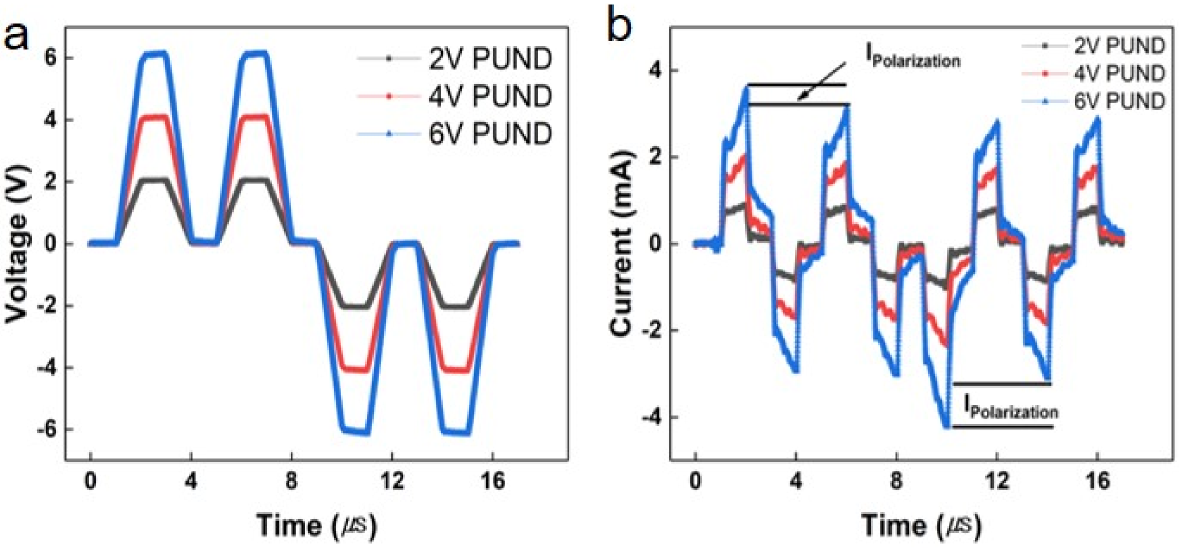

Figure S3. **PUND test and Polarization current for Pt/HfO$_2$/Pt device after annealed 600 °C and 30 min**. (a) Bias pulse wave setup for PUND test. (b) Current vs. pulse time plot of PUND test for Pt/HfO$_2$/Pt device after annealed 600 °C for 30 min.

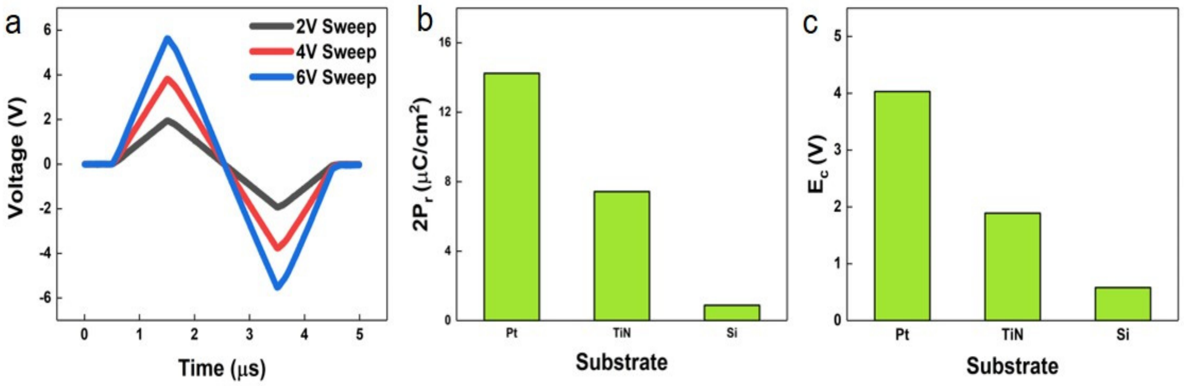

Figure S4. **Ferroelectric Properties for each device** (a) Bias pulse wave setup for drawing polarization curve. (b) Remanent polarization ($2P_r$) and (c) coercive field (E_c) data of HfO_2 film deposited on Pt, TiN, Si substrate at 6V sweeping.

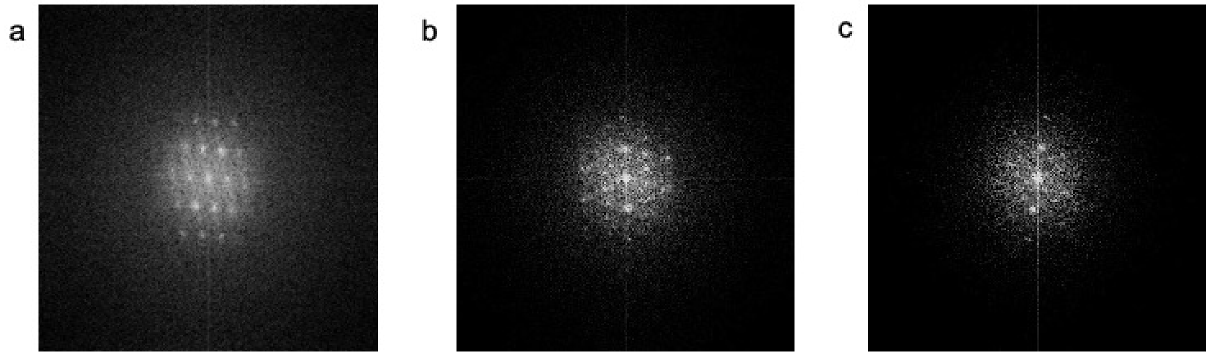

Figure S5. **FFT images** The low-resolution FFT images of local point in bulk HfO_2 film deposited on (a) Pt, (b) TiN, (c) Si and annealed at 600 °C for 30 min.

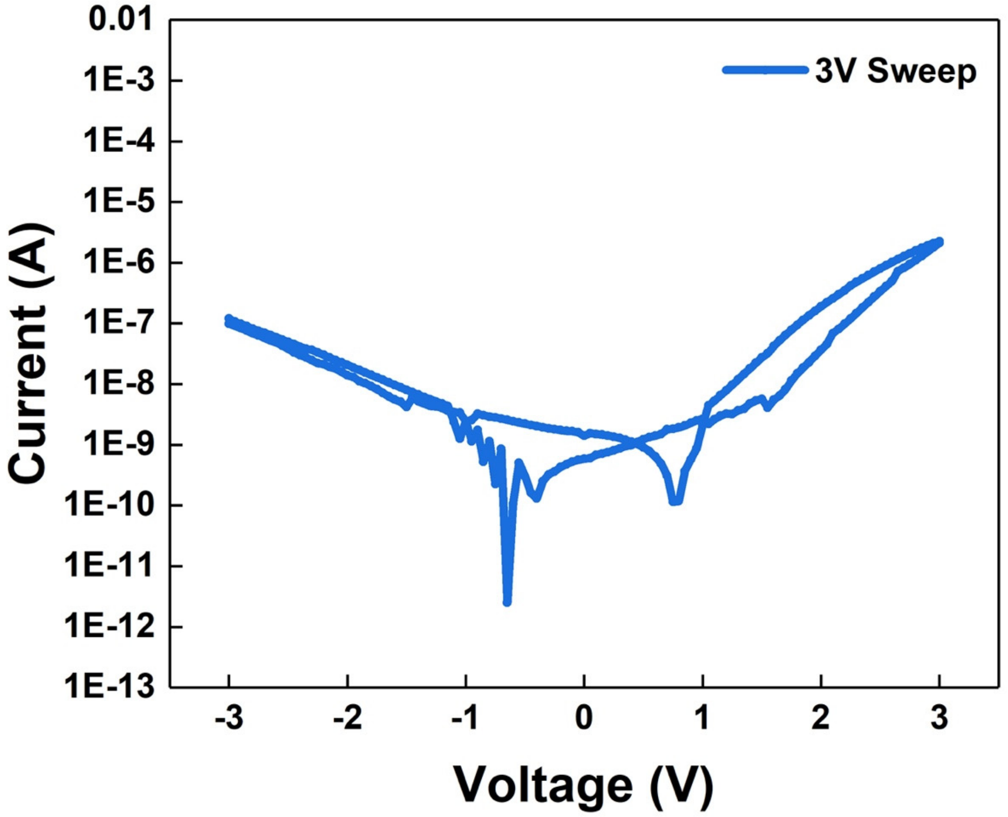

Figure S6. **I-V Curve** Representative switching current of HfO_2 film deposited on Pt Substrate and annealed at 600 °C for 30 min.

Table S1. XPS information for HfO_2 film deposited on Pt, TiN, Si and annealed at 600 °C for 30 min

Samples	O 1s (Hf_2O_3) [eV]<br>Area [%]	O 1s (V_o) [eV]<br>Area [%]	O 1s (HfO_2) [eV]<br>Area [%]	O 1s (Ti_2O_3) [eV]<br>Area [%]
Pt	532.78	531.85	530.9	
	27.71	7.76	64.53	
TiN	533.02	532.43	530.87	531.61
	33.51	6.1	45.99	14.4
Si	532.93	532.03	531.08	
	24.9	6	69.1	

Samples	Hf^{4+} $4f_{5/2}$ [eV]<br>Area [%]	Hf^{4+} $4f_{7/2}$ [eV]<br>Area [%]	Hf^{3+} $4f_{5/2}$ [eV]<br>Area [%]	Hf^{3+} $4f_{7/2}$ [eV]<br>Area [%]
Pt	19.34	17.74	18.76	17.14
	30.53	39.14	12.42	17.9
TiN	19.55	17.87	18.98	17.34
	33.38	38.41	13.04	15.17
Si	19.57	17.94	18.78	17.18
	36.76	47.33	5.48	10.43
